# Color Doppler Evaluation of Arterial Resistive Index in Infantile Hemangioma: A Useful Parameter to Monitor the Response to Oral Propranolol?

**DOI:** 10.3389/fped.2021.718135

**Published:** 2021-12-07

**Authors:** George Koshy Parapatt, Teresa Oranges, Guglielmo Paolantonio, Lucilla Ravà, Simona Giancristoforo, Andrea Diociaiuti, May El Hachem, Massimo Rollo

**Affiliations:** ^1^Interventional Radiology Unit, Department of Imaging, Bambino Gesù Children Hospital (IRCCS), Rome, Italy; ^2^Dermatology Unit, Department of Pediatrics, Meyer Children's Hospital, Florence, Italy; ^3^Unit of Clinical Pathways and Epidemiology, Bambino Gesù Children Hospital (IRCCS), Rome, Italy; ^4^Dermatology Unit and Genodermatosis Unit, Genetics and Rare Diseases Research Division, Bambino Gesù Children Hospital (IRCCS), Rome, Italy

**Keywords:** infantile hemangioma (IH), resistive index (RI), color Doppler, oral propranolol, ultrasound, radiologic evaluation, soft tissue tumor of infancy, vascular tumor

## Abstract

Infantile hemangioma (IH) is the most common benign vascular tumor in childhood. In more than 85% of all cases, IHs undergo spontaneous involution, but nearly 10–12% of IHs develop complications and require immediate therapy. Oral propranolol is currently the first-line treatment for IHs. Color Doppler ultrasound is the gold standard in the diagnosis of deep IH, and it is used to evaluate the morphological change and the modification of vascularization that occur during its evolution and treatment. To date, only few data in the literature described the changes of intralesional arterial resistive index (RI) during treatment with propranolol; particularly, some authors have shown an increase of intralesional arterial RI in IHs with clinical regression during treatment with propranolol. The objective of this paper is to evaluate the changes of RI of the intralesional arteries of the IHs during the treatment with oral propranolol. We retrospectively analyzed a total of 64 IHs in 60 patients treated with oral propranolol with a good clinical response. Gray-scale ultrasonography and color Doppler imaging were performed before and during the therapy. The intralesional RIs were measured before and during the treatment. For each lesion, we recorded the RI values, and then we calculated the mean RI value for any single lesion. We compared the mean RI value observed at the baseline with the mean RI value of the last detectable sampling at color Doppler. We also compared between them the mean RI values observed during intermediate ultrasound. The RI values were compared in 44 lesions, with at least two significant samplings of RI. In the 44 lesions compared, we did not find statistically significant variations in the mean RI values between the baseline control and the values recorded at the last post-treatment control. The time trend of mean RI values of the intermediate color Doppler analysis performed between the first pre-treatment control and the last measurable control did not show any statistically significant variation in the trend of mean RI values. Contrarily to what has been described by some authors, in our experience, we have not observed an increase of RI in IHs treated with oral propranolol.

## Introduction

Infantile hemangioma (IH) is a benign vascular tumor composed of proliferating endothelial-like cells (1–3). IHs are the most common soft tissue tumors in childhood (2, 4). IH is observed in 5 to 10% of infants (3–6). They occur most frequently in infants of female gender, in cases of premature and low birth weight, in placental anomalies, in aged mothers, and in twin pregnancies (6). IHs appear generally during the first weeks of life (3, 5). They mainly affect the most the head and neck region, followed by the trunk and the extremities (7, 8). Proliferation starts during the first weeks of life, reaching a peak at 3–6 months; then, it is followed by a spontaneous involution phase during the childhood period (5, 9). In more than 85% of all cases, IHs undergo spontaneous involution and are innocuous, but nearly 10–12% of IHs develop complications such as ulceration, functional impairment, and permanent disfigurement requiring immediate therapy (3, 4, 6, 9). In 2008, Léauté-Labrèze et al. described, for the first time, the efficacy of propranolol in the treatment of severe IH. Thereafter, extensive studies have confirmed the efficacy and safety of propranolol, and it is currently the first-line treatment for IHs (3, 10–12). Color Doppler ultrasound is the first-line investigation in suspected IH, and it is used to evaluate the morphological change and the modification of vascularization that occur during its evolution and treatment (2, 3, 11, 13). Although ultrasound examination is widely described as a consolidated method in the evaluation of IH (2, 3, 5, 14, 15), to our knowledge, few reports have assessed the sonographic changes of IHs during the treatment with propranolol (2, 13, 15, 16). Arterial resistive index (RI) is a semi-quantitative ultrasound Doppler parameter used to assess the vascular flow inside a lesion or organ. RI is an indicator of the activity of the vascular component of a tumor, and it is used to assess changes in intralesional vascular flow. The clinical observation of color change in IHs reflects the action of propranolol on intralesional vascular activity. Lower vascular activity within the tumor results in a change of RI values (5, 17–21). Contrarily to what has been described by some authors (5, 16, 21, 22) who have shown an increase of intralesional arterial resistive index in IHs during treatment with propranolol, these data have not been observed in our experience. The objective of this paper is to evaluate changes of RI of the intralesional arteries of IHs during the treatment with propranolol. Ultrasound examination is not performed as a routine at our institution for the evaluation of IHs; it is used for complex IHs only. Propranolol is administered at a dose of 2 mg/kg/day. In the treatment of IHs, this dose is the standard of care at our institution, except for PHACE syndrome and premature patients.

## Materials and Methods

We retrospectively analyzed a total of 64 IHs in 60 patients successfully treated with oral propranolol during 2013–2015 (Table 1). All patients had indication for treatment and came for observation at the outpatient vascular anomalies clinic of our pediatric hospital. Before the initiation of propranolol, each patient underwent a full medical history and clinical evaluation. All patients were treated with a dose of propranolol at 2 mg/kg/day. The IHs were located on the nose tip (Cyrano type), orbital–palpebral area, parotid and craniofacial region, trunk, and limbs. All patients underwent combined gray-scale and color Doppler ultrasound examinations before and during the propranolol therapy. Clinical evaluations were carried out on a monthly basis, as per international guidelines, together with monitoring of the main clinical parameters (glycemia, blood pressure, and heart beat); they were weighed to adjust the drug dosage and ensure adherence to treatment. In addition, during the treatment, ultrasound and color Doppler evaluation of the lesions were carried out. Ultrasound scans were performed before the start of propranolol and during the therapy until the disappearance of measurable intralesional spectral Doppler signals. Gray-scale ultrasonography and color Doppler imaging were scheduled at intervals of 1, 3, 6, 9, and 12 months. All ultrasound evaluations were performed by two interventional radiologists with experience in vascular anomalies. Both radiologists work alternately in the dermatology outpatient service, and the younger radiologist was trained by the older radiologist; the same patient was not assessed by both radiologists on the same day. In non-compliant infants, to minimize motion or crying artifacts, examinations were conducted while the patient was asleep whenever possible.

**Table 1:** Demographic and clinical data of all infantile hemangiomas (IHs).

	***n***
Total no. of IH	64
Avarage age at diagnosis (months)	3.5
Average age at onset of treatment (months)	5
Sex	
Male	19
Female	41
Location	
Nose tip	11
Periorbital	24
Parotid region	4
Craniofacial region	18
Trunk–limbs	7

Ultrasound examination and color Doppler evaluations were performed with a Philips CX-50 CompactExtreme ultrasound scanner (Philips Medical Systems Nederland B.V., Best, Netherlands) with a high-frequency (12–3 MHz) linear probe. In the evaluation of the vascular signals of IHs, we did not use the power Doppler to avoid excessive amplification of the flow signal.

Ultrasound scan was carried out using the standard preset for “surface organs” to assess the location and to establish the size and morphology of the lesions.

Color Doppler evaluation and resistive index (RI) analysis were performed using an “arterial” preset. A spectral analysis of vascular signals was obtained by placing a sample volume within intralesional arteries on color Doppler scans. RI was automatically measured by the scanner using the dedicated measurement tool, according to the formula:


$$RI=\frac{\left(PSV-EDV\right)}{PSV}$$

where PSV is the peak systolic velocity, and EDV is the end diastolic velocity (16, 20).

For each lesion, intralesional RI values were calculated at several points, according to the size of the lesion and the possibility of sampling. For each lesion, we recorded the RI values, and then we calculated the RI mean value for any single lesion. Intralesional RIs were measured before and during the treatment, until intralesional vascular signals were evident and detectable with color Doppler ultrasound. For each lesion, we compared the mean RI value observed at baseline with the value of the last detectable sampling signal at color Doppler. The disappearance of detectable intralesional vascular signals was observed at the ultrasound follow-up at 1-, 3-, and 6-month intervals. In addition, to evaluate the time trend of the mean RI values, for each lesion, we also compared the mean RI values observed during intermediate color Doppler ultrasound with the first pre-treatment examination and the last measurable vascular signal at color Doppler.

### Statistical Methods

Demographic and clinical data were reported as counts and percentages for categorical variables and as median and interquartile range for continuous variables. Chi-square test or Fisher exact test was used in order to compare proportions and Wilcoxon test to compare medians. Differences across time were evaluated by McNemar test for matched categorical data, while Wilcoxon signed-rank test or Friedman test (*t*-test or ANOVA) was used for continuous dependent variables. Mixed-effect models were used in order to evaluate the time trend of average RI. This kind of model allow repeated-measures data when the number of observations for each individual is not the same. All analyses were performed with Stata 13.1 (StataCorp LLC, College Station, TX). A *p* <0.05 was considered statistically significant.

## Results

**Figure 1:**
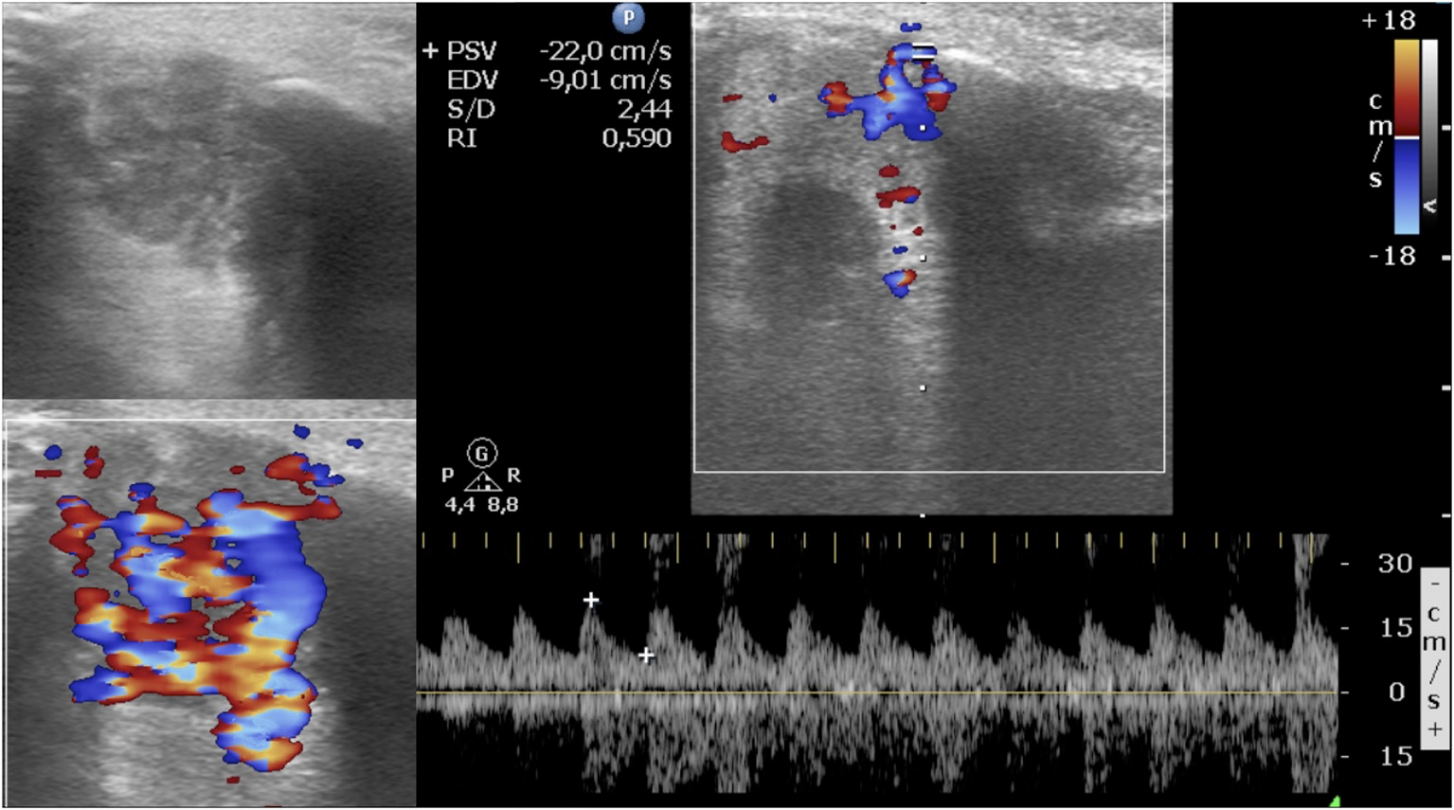
Periorbital infantile hemangioma. Baseline ultrasound images show a circumscribed mostly hypoechoic mass; at color Doppler, the lesion is highly vascularized, and the resistive index is 0.59.

**Figure 2:**
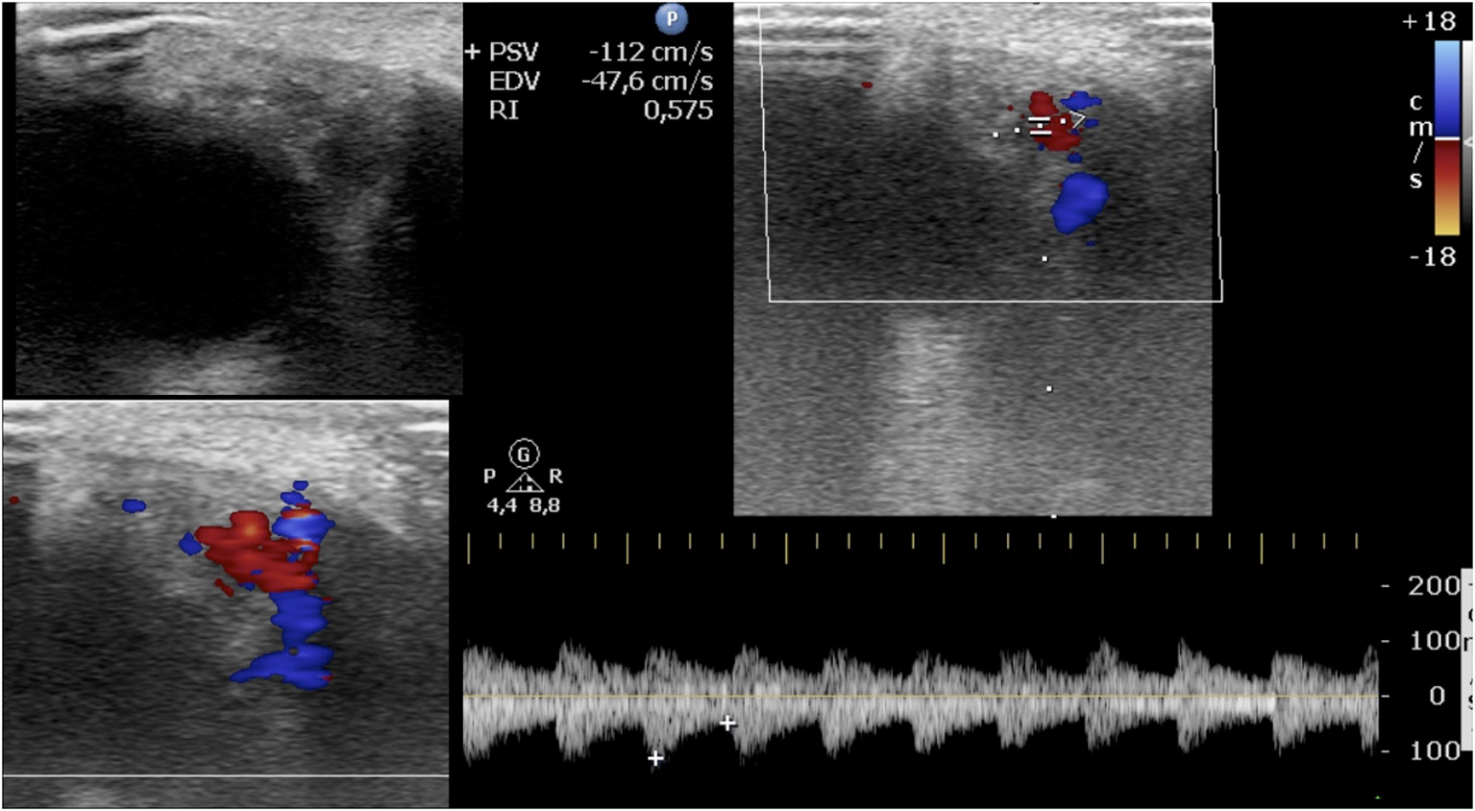
The same infantile hemangioma of Figure 1. Latest ultrasound evaluation with detectable color Doppler signals after 3 months of oral propranolol. Post-treatment images show a smaller mass with heterogeneous echogenicity; at color Doppler, the vascularization is reduced, and the resistive index is 0.57.

A total of 64 IHs in 60 patients were retrospectively analyzed. Four children had two IHs. All IHs had a good clinical response to oral propranolol. A good clinical result is defined as a complete or almost complete or partial regression of IH. The infants were 19 male and 41 female. The age at diagnosis was between 0.9 and 8.3 months, with an average age at diagnosis of 3.5 months. The average age of initiation of treatment was 5 months (Tables 1, 2). Twenty out of 64 lesions were excluded from the evaluation of RI values because, at the ultrasound examination after 1 month of therapy, they did not show significant vascular signals for sampling. The RI values where then compared in 44 lesions with at least two significant samplings of RI and evaluated until the disappearance of the color Doppler signals. Among these 44 lesions, 24 were superficial IHs, 13 mixed his, and seven deep IHs. In 44 lesions, we recorded the values sampled in each IH and calculated the mean RI value. We compared the mean RI values at baseline with the mean RI values at the last control during treatment, in which it was possible to sample the vascular signals. The last ultrasound examination in which it was possible to detect and sample RI values was the evaluation at the first month after therapy in 14 lesions, at the third month of therapy in nine lesions, and at the sixth month of therapy in 21 lesions. In the 44 lesions compared, we did not find statistically significant variations in the mean RI values between the baseline control (0.57) and the values recorded at the last post-treatment control, in which there were appreciable vascular signals (0.58) (Table 3) (Figures 1, 2).

**Table 2:** Distribution of patients and type of IHs during different controls over time.

	**No. of patients**	**No. of patients**	**No. of patients**	**No. of patients**
	**at baseline**	**at month 1**	**at month 3**	**at month 6**
Total	44	44	30	21
Superficial IH	24	24	14	10
Mixed IH	13	13	11	8
Deep IH	7	7	5	3

**Table 3:** RI values at the baseline and at the last post-treatment control.

**Variable**	**n**	**Mean**	**S.D**.	**Min**	**.25**	**Mdn**	**.75**	**Max**
max RI baseline	44	0.61	0.10	0.38	0.54	0.62	0.69	0.88
min RI baseline	44	0.52	0.10	0.34	0.45	0.52	0.58	0.75
mean RI baseline	44	0.57	0.09	0.38	0.51	0.58	0.62	0.75
max RI last control	44	0.62	0.09	0.42	0.56	0.60	0.68	0.82
min RI last control	44	0.54	0.09	0.40	0.47	0.53	0.59	0.82
mean RI last control	44	0.58	0.08	0.41	0.52	0.58	0.63	0.82

*S.D., standard deviation; Mdn, median; min: minimum; max, maximum; 25, 75, percentiles*.

The mixed-effect model did not show any statistically significant time trend in average RI values of the intermediate Doppler analysis.

## Discussion

Oral propranolol is recommended as the first line for treatment of IHs (4, 12). Since 2008, the beneficial effects of the use of this treatment have been extensively described. Multiple studies demonstrated the clinical effectiveness of propranolol in terms of color changes, palpable softening of the hemangiomas, and reduction of the area of the IH (2–4, 12).

Ultrasound imaging has many advantages over clinical observations during the treatment. It allows an objective documentation of dimensional and anatomical changes of IHs during oral propranolol administration (5). As already stated by some authors, gray-scale and color Doppler ultrasound is a widely available and non-invasive method that not only provides accurate measurements of the dimensions of IHs but also demonstrates their relationships with the surrounding structures and tissues and gives information about the echogenicity of the lesions (2, 5, 16, 21).

Color Doppler ultrasound allows evaluation of the vascularization of IHs by detecting intralesional arterial and venous blood flow signals, blood flow velocities, and arterial RI (3, 18, 23). Other reports have already described and demonstrated changes seen at ultrasound scan regarding dimensions, volume, and echogenicity of hemangiomas during the treatment with oral propranolol (2, 8, 15, 16). In our population, a dimensional reduction of IHs treated with oral propranolol has been observed clinically, but it was not specifically evaluated in this study. Some authors reported previously the correlation between good clinical response to propranolol and the dimensional reduction observed on ultrasound. Kutz et al. described a positive clinical response of the IHs under oral propranolol treatment and documented on color Doppler ultrasound. Interestingly, they reported, as well as other authors, a reduction of the volume of the lesion, its thickness, and its longitudinal and transverse diameters (2, 5, 8, 15, 16). To date, to our knowledge, collectively few publications assessed the ultrasound changes in IHs treated with oral propranolol. Particularly, very few manuscripts had described the trend of intralesional RI values during treatment (2, 5, 15, 16, 21, 22). On color Doppler scan, vascularization can be evaluated by using RI, which measures the resistance of blood flow. The velocity curve of an artery is characterized by a maximum peak and a minimum peak and by a diastolic velocity; RI is a semi-quantitative measurement obtained using these parameters (20). As already defined, RI is the difference between peak systolic and end diastolic arterial velocities, divided by the peak systolic velocity. Since RI is the result of a proportion of velocities measured at the same Doppler angle, the latter is eliminated. This makes RI particularly useful in assessing flow rate variations with unfavorable Doppler angles (20). In addition, this leads to avoiding angle correction on Doppler scan and reduces operator errors; therefore, in the pediatric setting, this is an important favorable factor. Unlike other authors, in our color Doppler evaluation of treated IHs, we did not consider arterial flow velocity (2, 5, 15, 16). Evaluation of flow velocity needs the calculation of the angle of incidence, which could be a problem when performing ultrasound evaluation in a non-sedated pediatric population and would lead to increased operator error (20). In fact, infants do not collaborate during the ultrasound examination, so the evaluation of IHs should be performed in a timely manner. Regarding intralesional RI values, Talaat et al. described in 50 infants a significant increase of RI values on ultrasound examination in treated IHs with clinical regression. Similarly, Shi et al., as well as Chang et al., described the responses of IHs to propranolol in 31 patients and observed significantly higher values of RIs during the treatment than before treatment. Moreover, Ginguerra et al. reported that an increase of arterial RI on Doppler ultrasound correlated to clinical involution in seven patients during the treatment with propranolol. In our patients, the trend of RI values was different compared to previous reports. In the 44 cases studied, we did not find significant variations in the mean RI values between the pre-treatment and the values recorded at the last post-treatment control with detectable and measurable vascular signals. The mean RI value at the baseline control was 0.57 and at the last post-treatment was 0.58. Similarly, the time trend of the mean RI values of the intermediate Doppler evaluation between the pretreatment baseline control and the last measurable control did not show any significant variation. Contrarily to what has been stated by other authors (2, 5, 16, 21), we observed a depletion of vascularization in IHs without an increase in RI, and the arterial RI values remain essentially stable. The mean RI values we found are comparable to the RI value (0.59) observed by Dubois et al. in untreated IHs (18, 24). Our study confirmed that IHs are low-resistance vascular tumors as reported by other authors. Color Doppler shows arterial flow with low resistance with relatively high velocities in IHs (24, 25). A soft tissue mass with high arterial RI should raise suspicion that it could not be an IH. In fact, Dubois already stated that other benign or malignant tumoral lesions (e.g., sarcoma, neuroblastoma, myofibromatosis, tufted angioma, hemangiopericytoma, and others) should be ruled out in case of a soft tissue mass showing high RI values (18, 24). In addition, the works by Chiou et al. and Rimondi et al. also associate low internal arterial resistances as a characteristic of infantile hemangiomas, and both found moderate and high arterial RI values in aggressively behaving lesions (24, 26, 27).

Considering our results and the literature data, we suggest that the stability of low RI values in treated IHs is an important factor in the pediatric setting, and RI measurement may represent a key for differential diagnosis between IH and other soft tissue lesions (24, 25). In addition, our results, unlike those of other authors (21), do not support the concept that RI can be a valid radiological complement to clinical observation in the decision to discontinue therapy.

Unlike the cited works (5, 16, 21, 22), our study places exclusive emphasis on the importance and trend of RI in hemangiomas under treatment. RI gives information on the vascular resistance of the lesion and therefore on the perfusion characteristics of the hemangioma, and in particular, it characterizes the arterial flow of the lesion (20, 23, 24, 26). On the basis of our experience and the results from the observation of our cases, we believe, also according to the literature, that RI can contribute to specifically characterize hemangiomas under treatment with propranolol (18, 19, 23, 24, 26, 27). In infantile hemangioma which, in its evolution, significantly modifies ultrasound morphological features, echostructure, and vascular density, we observe instead an RI trend characterized by low resistances.

To evaluate intralesional flow variations, we considered the RI parameter to be more reliable than Doppler velocity variations, which is influenced by the angle of insonation (17, 20).

The limitation of this study is the absence of controls, i.e., IHs without propranolol treatment, so we could not evaluate RI changes in infantile hemangiomas undergoing natural involution. However, in agreement with the data of Dubois, we expect the same RI trend in IHs with spontaneous involution (18, 24). Moreover, we are continuing to use color Doppler and RI measurement in our outpatient evaluation of small patients with IHs, thus, we are confident that future data can address this limitation. In conclusion, color Doppler ultrasound permits a valid and objective non-invasive quantification of the response in IHs during propranolol therapy, particularly of the vascularization of IHs. We detected in our treated patients a depletion of vascularization of the IHs and a stability of RI values, contrarily to what has been stated by some authors. Thus, we believe that RI cannot be used as a parameter to evaluate the response to treatment. In addition, this study confirmed that IHs are low-resistance vascular tumors. Finally, we consider that RI may represent a tool in distinguishing infantile hemangiomas from other soft tissue masses with aggressive behavior.

## Data Availability Statement

The raw data supporting the conclusions of this article will be made available by the authors, without undue reservation.

## Ethics Statement

Ethical review and approval was not required for the study on human participants in accordance with the local legislation and institutional requirements. Written informed consent from the participants' legal guardian/next of kin was not required to participate in this study in accordance with the national legislation and the institutional requirements.

## Author Contributions

GKP and GP wrote the article and have contributed equally to this work and share first authorship. TO and SG contributed in drafting the article. LR performed the statistical analysis and data interpretation. MH and AD have contributed in drafting the article and contributed to the final version. MR is the last author, conceived the original idea and supervised the project, and contributed to all phases of the article. All authors contributed to the article and approved the submitted version.

## Conflict of Interest

The authors declare that the research was conducted in the absence of any commercial or financial relationships that could be construed as a potential conflict of interest.

## Publisher's Note

All claims expressed in this article are solely those of the authors and do not necessarily represent those of their affiliated organizations, or those of the publisher, the editors and the reviewers. Any product that may be evaluated in this article, or claim that may be made by its manufacturer, is not guaranteed or endorsed by the publisher.
